# Autologous Iliac Crest Bone Graft After Anterior Cruciate Ligament Reconstruction Failure in Patients With Both Femoral and Tibial Tunnel Enlargement

**DOI:** 10.1002/atn2.70248

**Published:** 2026-09-12

**Authors:** Aoqiu Wu, Yuchen He, Shiqi Xiang, Chang Xiong, Weihong Zhu

**Affiliations:** ^1^ Department of Orthopaedics The Second Xiangya Hospital, Central South University Changsha, Hunan China

## Abstract

Tunnel enlargement is a common challenge in revision anterior cruciate ligament reconstruction and compromises graft fixation and biological integration. Traditional 2‐stage revision procedures prolong treatment duration and increase patient morbidity. We describe a technique using autologous cancellous bone harvested from the ipsilateral iliac crest to restore femoral and tibial tunnel bone stock after anterior cruciate ligament reconstruction failure. This technique focuses on managing tunnel enlargement and prepares the tunnels for subsequent anterior cruciate ligament revision. Arthroscopic impaction of autologous bone restores tunnel geometry and provides a favorable biological environment for fixation. In selected patients, this approach may shorten rehabilitation time and eliminate the need for staged surgery.

## Graphical abstract

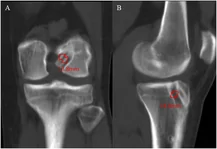

**VIDEO 1:** Arthroscopic inspection showing rupture of the previously reconstructed anterior cruciate ligament and marked enlargement of both the femoral and tibial tunnels. Residual graft fibers, fibrous scar tissue, and tunnel debris are thoroughly debrided using a motorized shaver and curette until healthy bleeding cancellous bone is exposed. The size, orientation, and containment of the femoral and tibial tunnels are assessed arthroscopically. Autologous cancellous bone is harvested from the ipsilateral iliac crest through a small oblique incision. The bone block is trimmed and contoured to match the diameter of the enlarged tunnels. A central longitudinal tunnel is created within the bone block, and passing sutures are threaded through the graft to facilitate controlled arthroscopic delivery. Under arthroscopic visualization, the suture‐loaded bone block is introduced into the femoral tunnel along a guide wire. Gentle traction on the sutures allows accurate positioning of the graft within the tunnel. Progressive impaction is performed using a bone tamp to achieve firm contact between the graft and the tunnel walls while preserving a central channel for subsequent graft passage. The same technique is applied to the tibial tunnel. The autologous iliac crest bone graft is advanced into the tibial tunnel using the passing sutures and is sequentially compacted with a bone tamp to restore tunnel geometry and bone stock. Arthroscopic inspection confirms uniform tunnel filling and stable graft impaction without displacement. Video content can be viewed at https://doi.org/10.1002/atn2.70248.

Revision anterior cruciate ligament (ACL) reconstruction is frequently complicated by femoral and tibial tunnel enlargement resulting from graft micromotion, osteolysis, or fixation failure.^1^ Enlarged tunnels reduce bone stock and compromise fixation strength, making revision surgery technically demanding. Traditionally, a 2‐stage approach with initial bone grafting followed by delayed reconstruction has been recommended. Although effective, this strategy prolongs recovery and increases patient burden.^2^, ^3^

Autologous cancellous bone grafting provides osteogenic potential and avoids risks associated with allografts.^4^ When securely impacted, autologous bone grafting may restore tunnel geometry and permit immediate ligament reconstruction.^5^ This article describes a reproducible technique using autologous iliac crest bone grafting specifically for the management of femoral and tibial tunnel enlargement after ACL reconstruction failure.

## SURGICAL TECHNIQUE

### Indications and Contraindications

Indications include ACL deficiency with femoral and/or tibial tunnel enlargement ≥14 mm confirmed by computed tomography, normal limb alignment, and patient compliance with rehabilitation.

Contraindications include active infection, severe malpositioned tunnels requiring osteotomy, open physes, and massive bone loss preventing stable graft impaction.

#### Equipment and Implants

The following equipment and implants were used in the described technique:

• Iliac crest bone graft harvest was performed using a standard autograft harvesting saw and curettes (medical‐grade orthopaedic instruments).
• ACL fixation devices included the ToggleLoc Device with ZipLoop Technology (Zimmer Biomet, Warsaw, IN), a knotless femoral fixation system that facilitates adjustable suspensory fixation for ACL grafts and allows tensioning from the femoral side.
• Interference fixation used Biosteon interference screws (manufactured by Biocomposites and distributed by Stryker, Kalamazoo, MI), composed of calcium composite material for graft fixation within the tunnels.
• Additional fixation options compatible with the described technique include BTLoop adjustable fixation system (SBM, 65100 Lourdes, France), which provides cortical suspensory fixation for soft tissue grafts.
• Arthroscopic ACL instruments and guides were provided by Arthrex Instrument Sets (Arthrex, Naples, FL), including cannulated reamers, drill guides, push‐rods, and rasp handles to facilitate tunnel preparation and graft impaction.

All instruments were sterilized according to institutional protocols prior to use and were selected based on tunnel diameter, patient anatomy, and surgeon preference.

### Diagnostic Arthroscopy and Tunnel Preparation

**FIGURE 1:**
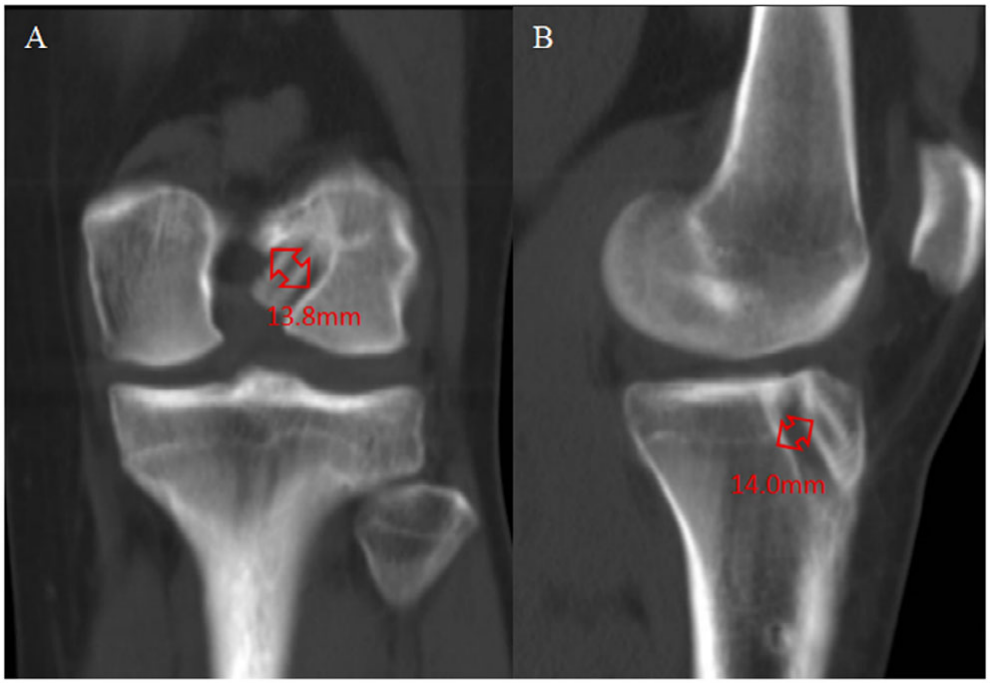
Preoperative CT assessment of femoral and tibial tunnel enlargement. Preoperative CT scans of the right knee in a supine patient. (A) Sagittal CT image showing femoral tunnel enlargement (arrow indicates tunnel), with a diameter of approximately 13.8 mm prior to revision. (B) Coronal CT image showing tibial tunnel widening (arrow indicates tunnel), measuring 14.0 mm. These findings guide surgical planning for autologous bone grafting. Orientation: sagittal and coronal planes; lateral view indicated with arrowhead. (CT, computed tomography.)

The patient is positioned supine under general anesthesia with a pneumatic tourniquet. Standard anteromedial and anterolateral portals are established. Arthroscopy confirms graft failure and tunnel enlargement. Residual graft tissue and fibrous debris are removed until bleeding cancellous bone is exposed. Tunnel diameter and orientation are assessed and correlated with preoperative computed tomography findings (Figure 1).

### Autologous Iliac Crest Bone Graft Harvesting

**FIGURE 2:**
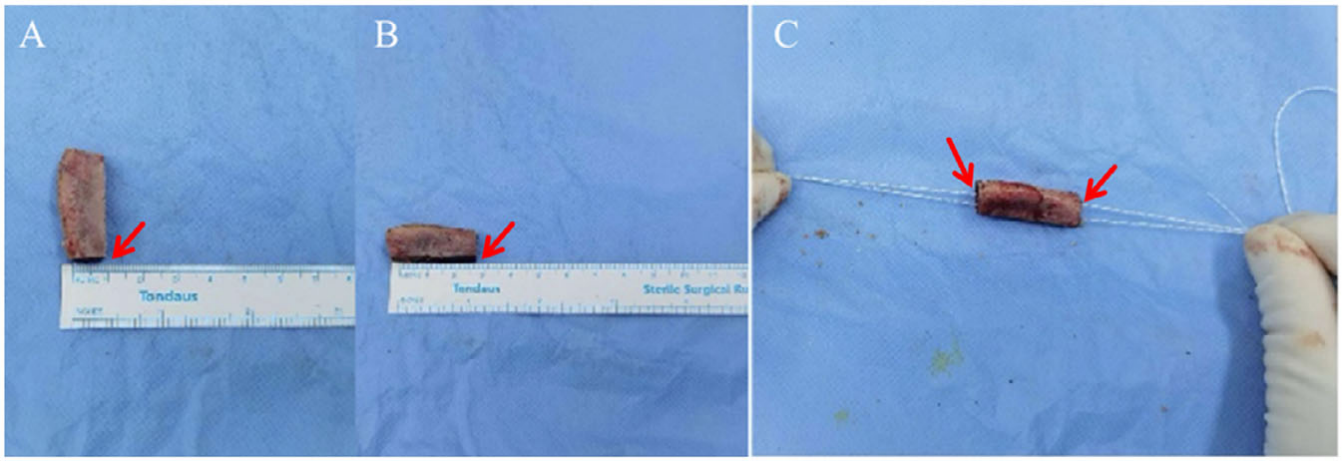
Preparation of the autologous iliac crest bone block. Harvested iliac crest bone from the ipsilateral side of a supine patient. (A) Rectangular cancellous bone block with intact cortical margins. (B) Measurement of bone block length and width to match femoral and tibial tunnel dimensions (arrows indicate measurement points). (C) Sutures passed through the central axis to facilitate controlled insertion during arthroscopic impaction (arrow indicates suture passage).

Iliac crest harvest was carried out using a sterile bone harvesting saw and curettes (orthopaedic instrument set), ensuring optimal cancellous bone collection for tunnel grafting. Arthroscopic portal creation and cannulation employed the Arthrex ACL ToolBox and cannulas (Arthrex, Naples, FL), facilitating accurate positioning and minimal soft tissue disruption. A 3‐cm oblique incision is made over the ipsilateral iliac crest. A cancellous bone block is harvested and trimmed to match the diameter of the enlarged tunnels. A central longitudinal channel is created, and high‐strength sutures are passed through the graft to facilitate arthroscopic delivery (Figure 2).

### Bone Grafting

**FIGURE 3:**
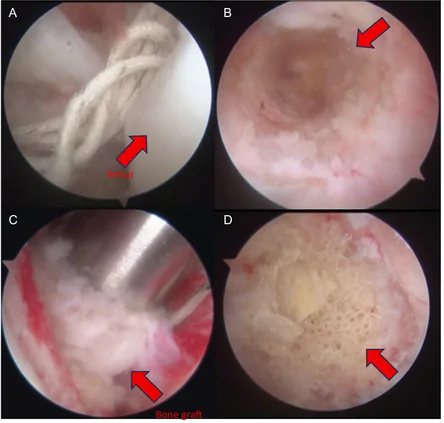
Arthroscopic management of tunnel enlargement and bone graft insertion. Arthroscopic view of the right knee in supine position using standard anteromedial and anterolateral portals. (A) Femoral tunnel enlargement with residual graft fibers prior to debridement (arrow marks tunnel). (B) Enlarged femoral and tibial tunnels after debridement (arrows indicate tunnels). (C) Insertion of suture‐loaded iliac crest bone graft into tunnels (arrow indicates graft). (D) Final arthroscopic assessment confirming tunnel restoration (arrow indicates filled tunnel).

**FIGURE 4:**
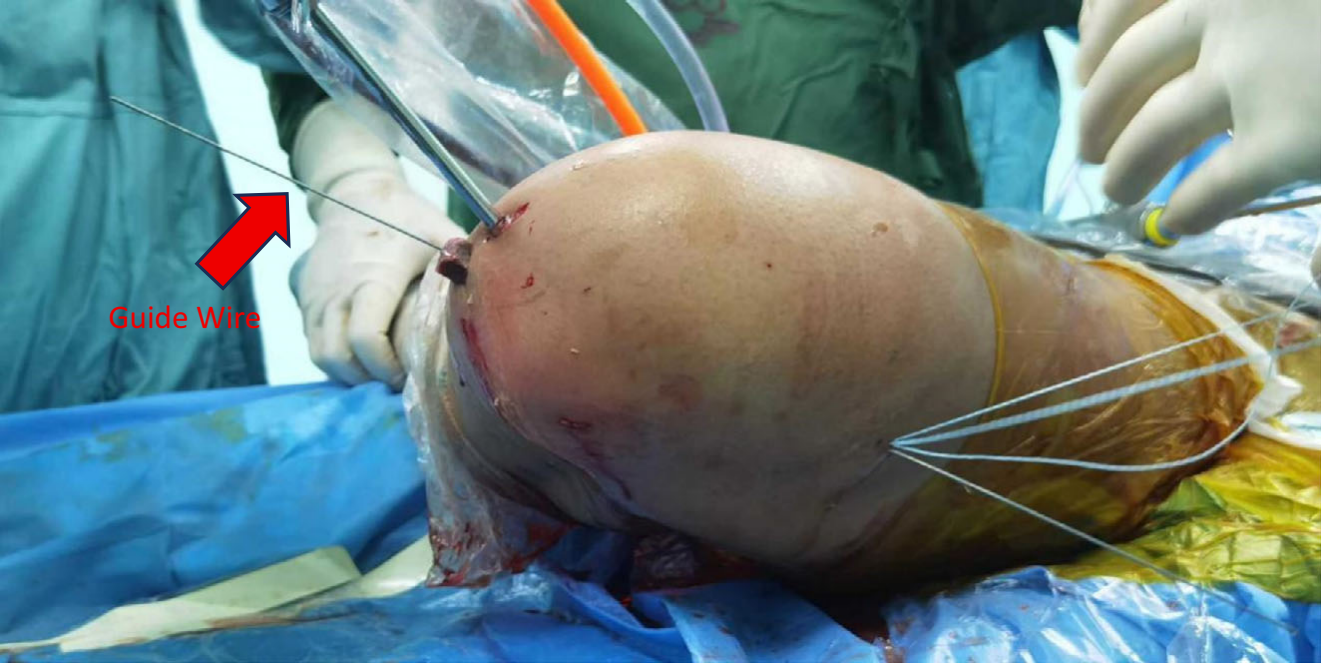
Insertion of the autologous bone block via guide wire. Arthroscopic view of right knee with patient supine. A 1.5‐mm flexible guide wire (arrow) is used to guide the autologous bone graft into the femoral tunnel, ensuring precise placement under visualization. A bone tamp is used to impact the graft (arrowhead).

**FIGURE 5:**
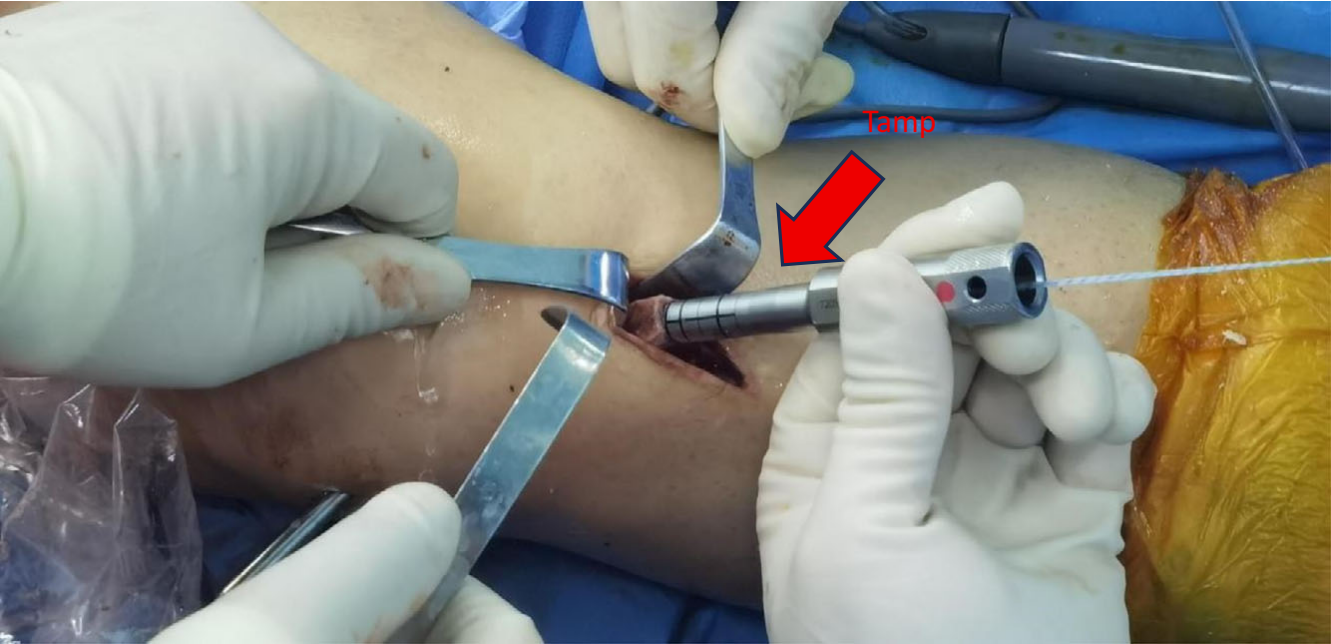
Impaction and fixation of the bone block in tibial tunnel. Right knee in supine position, tibial tunnel visualization via anteromedial portal. Prepared iliac crest bone graft is impacted using a bone tamp (arrow indicates tamp direction) until secure fixation and restoration of tunnel geometry are achieved.

A flexible guide wire is introduced into the femoral tunnel. The suture‐loaded bone graft is advanced along the guide wire and progressively impacted with a bone tamp until firm circumferential contact with the tunnel wall is achieved (Figures 3 and 4). The same technique is applied to the tibial tunnel, with sequential impaction until the tunnel is filled and stable fixation is achieved (Figure 5). Final arthroscopic inspection confirms restoration of tunnel morphology. This procedure restores the bone stock of the tunnels and prepares them for future ACL reconstruction.

### Assessment of Tunnel Restoration

**FIGURE 6:**
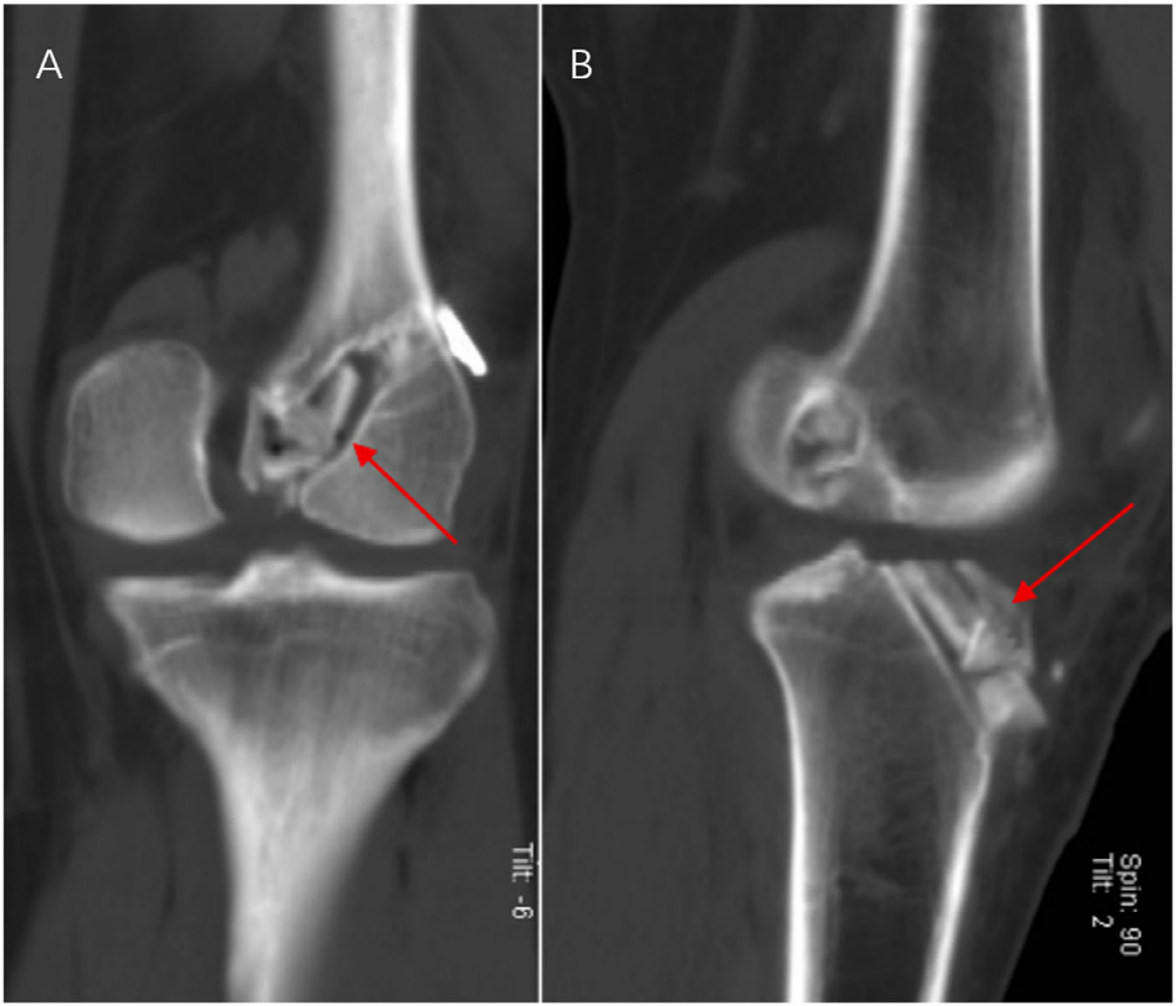
Postoperative CT evaluation of femoral and tibial tunnels. Postoperative CT of right knee in supine position. (A) Femoral tunnel showing complete graft impaction and restored diameter (arrow indicates tunnel). (B) Tibial tunnel showing full filling with autologous bone and restored morphology (arrow indicates graft location). Orientation: sagittal and coronal planes; arrows highlight grafted tunnels. (CT, computed tomography.)

**FIGURE 7:**
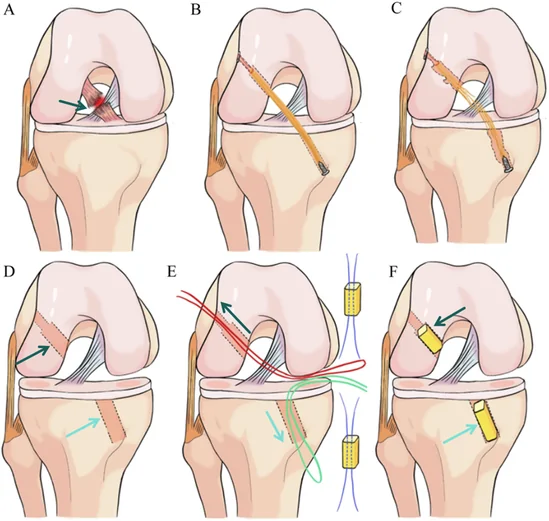
Simplified surgical workflow of single‐stage autologous bone grafting for revision ACL reconstruction. Surgical workflow of autologous iliac crest bone grafting for femoral and tibial tunnel enlargement in ACL revision. Supine patient, right knee. (A) Primary ACL rupture (arrow). (B) Initial ACL reconstruction. (C) Graft rupture after reconstruction. (D) Enlargement of femoral and tibial tunnels (arrows indicate tunnels). (E) Passage of suture‐loaded bone block through tunnels under arthroscopic guidance (arrow indicates graft direction). (F) Implantation of bone graft within tunnels to restore bone stock, preparing for definitive ACL reconstruction (arrow indicates graft placement). (ACL, anterior cruciate ligament.)

After completion of bone grafting, arthroscopic reassessment confirmed uniform filling of both tunnels. Postoperative computed tomography is used to confirm graft impaction and restoration of tunnel diameter (Figure 6). A schematic overview of the single‐stage workflow is shown in Figure 7.

## DISCUSSION

Tunnel enlargement compromises fixation strength and graft integration in revision ACL reconstruction. Bone grafting remains the most reliable method for restoring bone stock. Autologous iliac crest bone grafting effectively restores tunnel geometry and bone stock, facilitating future ACL reconstruction and optimizing graft fixation conditions.^5^ This technique should be considered a preparatory step for ACL revision in cases of tunnel enlargement, rather than a 1‐stage ACL revision procedure.^6^, ^7^

Autologous iliac crest bone provides predictable incorporation and excellent biological potential.^8^, ^9^ When tunnel containment and graft impaction are adequate, immediate ligament reconstruction using autologous or synthetic grafts is feasible.^10^, ^11^, ^12^, ^13^ However, careful patient selection and precise graft sizing are essential.^14^ In cases with poor bone quality or massive tunnel defects, a staged approach remains appropriate. Advantages, pitfalls, and limitations of this arthroscopic technique are summarized in Tables 1 and 2. The surgical procedure is shown in Video 1.

**TABLE 1:** Pearls and Pitfalls

Pearls	Pitfalls
Autologous iliac crest bone provides strong osteogenic potential	Inadequate debridement compromises graft‐bone contact
Accurate graft‐tunnel size matching ensures effective tunnel filling	Oversized grafts may impede tunnel passage
Graft edge trimming facilitates smooth arthroscopic delivery	Undersized grafts may result in insufficient tunnel filling

**TABLE 2:** Advantages and Disadvantages

Advantages	Disadvantages
Provides excellent osteogenic potential and promotes tunnel healing	Technically demanding with a steep learning curve
Restores tunnel geometry and improves future graft fixation conditions	Inaccurate graft sizing may compromise fixation
May reduce overall treatment duration compared with staged procedures	Iliac crest harvesting may cause donor‐site morbidity
Arthroscopic technique minimizes soft tissue disruption	Extremely large tunnel defects may still require staged revision surgery

Contemporary fixation device options enhance stability and may influence tunnel biology. For instance, adjustable suspensory systems such as ToggleLoc with ZipLoop Technology provide reproducible fixation across a range of tunnel lengths (Zimmer Biomet), whereas composite interference screws like Biosteon (Biocomposites/Stryker) offer osteoconductive fixation in graft integration.

## DISCLOSURES

The authors (A.W., Y.H., S.X., C.X., W.Z.) declare that they have no known competing financial interests or personal relationships that could have appeared to influence the work reported in this article.
